# Global analysis of the AP2/ERF gene family in rose (*Rosa chinensis*) genome unveils the role of *RcERF099* in Botrytis resistance

**DOI:** 10.1186/s12870-020-02740-6

**Published:** 2020-11-23

**Authors:** Dandan Li, Xintong Liu, Lizhe Shu, Hua Zhang, Shiya Zhang, Yin Song, Zhao Zhang

**Affiliations:** 1 Beijing Key Laboratory of Development and Quality Control of Ornamental Crops, Department of Ornamental Horticulture, China Agricultural University, Yuanmingyuan Xilu 2, Beijing, 100193 China; 2 College of Agronomy, Northwest A&F University, Yangling, 712100 Shaanxi China; 3 Beijing Key Laboratory of Greening Plants Breeding, Beijing Institute of Landscape Architecture, Beijing, China

**Keywords:** *Rosa* sp., AP2/ERF gene family, *Botrytis cinerea*, Virus-induced gene silencing

## Abstract

**Background:**

The AP2/ERFs belong to a large family of transcription factors in plants. The AP2/ERF gene family has been identified as a key player involved in both biotic and abiotic stress responses in plants, however, no comprehensive study has yet been carried out on the AP2/ERF gene family in rose (*Rosa* sp.), the most important ornamental crop worldwide.

**Results:**

The present study comprises a genome-wide analysis of the AP2/ERF family genes (*RcERFs*) in the rose, involving their identification, gene structure, phylogenetic relationship, chromosome localization, collinearity analysis, as well as their expression patterns. Throughout the phylogenetic analysis, a total of 131 *AP2/ERF* genes in the rose genome were divided into 5 subgroups. The *RcERFs* are distributed over all the seven chromosomes of the rose, and genome duplication may have played a key role in their duplication. Furthermore, Ka/Ks analysis indicated that the duplicated *RcERF* genes often undergo purification selection with limited functional differentiation. Gene expression analysis revealed that 23 *RcERFs* were induced by infection of the necrotrophic fungal pathogen *Botrytis cinerea*. Presumably, these *RcERFs* are candidate genes which can react to the rose’s resistance against *Botrytis cinerea* infection. By using virus-induced gene silencing, we confirmed that *RcERF099* is an important regulator involved in the *B.cinerea* resistance in the rose petal.

**Conclusion:**

Overall, our results conclude the necessity for further study of the AP2/ERF gene family in rose, and promote their potential application in improving the rose when subjected to biological stress.

**Supplementary Information:**

The online version contains supplementary material available at 10.1186/s12870-020-02740-6.

## Background

Transcription factors are important regulators of the expression of various inducible genes in plants, and play an indispensable role in plant growth, development, stress response, as well as pathogen defence [1]. Transcription factors usually comprise a nuclear localization signal, a DNA binding domain, a transactivation domain, as well as an oligomerization site. These domains determine the subcellular localization, cis-regulatory elements binding, and the regulating function of transcription factors [2].

The AP2/ERF superfamily is one of the largest transcription factor gene family in plants, wherein a total of 147 AP2/ERF family members have been identified in Arabidopsis. The AP2/ERF gene family consists of the AP2/ERF domain comprising 60 to 70 amino acids, and recognizes the cis-regulatory element GCC box or DRE elements which regulate the reaction of target genes [3]. The AP2/ERF gene family can be further categorized into five subfamilies, to example ERF, AP2 (APETALA2), DREB (dehydration-responsive element binding), RAV (related to ABI3/VP1) and Soloist [4–6]. The AP2/ERFs that regulate growth and development throughout the plant’s life cycle have been detected. The AP2/ERFs also play a very important role when the plant is exposed to abiotic stresses, such as dehydration, salinity, low temperature or heat stress. For example, transgenic Arabidopsis that overexpresses *AtERF4* is more sensitive to drought stress and has a lower resistance to Sodium chloride [7]. In addition, overexpressing the *RAP2.6* gene (*RELATED TO AP2.6*, encodes an ERF transcription factor) results in a sensitive phenotype to ABA (Abscisic Acid) and salt/osmotic stress during germination and the early growth stage of Arabidopsis [8].

More importantly, the AP2/ERF gene family is one of the transcription factors considered to be involved in plant defence responses against various phytopathogens [9–12]. For example, the transcript of *ERF1* is induced significantly subsequent to the inoculation of necrotrophic fungi *Botrytis cinerea*, and overexpression of *ERF1* in Arabidopsis enhanced its resistance to both *B. cinerea* and *Plectosphaerella cucumerina* [13]. Overexpressing *ERF5* or *ERF6* also increased resistance to *B. cinerea* in Arabidopsis, and the *erf5 erf6* double mutant showed a significant increase in susceptibility [14].

Rose is the most popular ornamental crop and accounts for over 30% of total cut-flower sales worldwide [15]. However, the flower is a fragile organ and transportation over long distances causes rose flowers to be affected by post-harvest diseases such as gray mold caused by *B. cinerea*. The function of AP2/ERF transcription factors in disease resistance has been characterized in model plants Arabidopsis as well as many other plant species. However, no rose AP2/ERF family genes involved in disease resistance have yet been identified.

Recently, we performed a de novo RNA-Seq analysis of rose petals infected by *B. cinerea*. This transcriptome study revealed a large number of rose genes, including AP2/ERF family transcription factors, were significantly up-regulated and implied their involvement of resistance against *B. cinerea* [16]. In the present study, genome-wide identification and analysis of the AP2/ERF gene family in the rose were carried out. By using virus-induced gene silencing (VIGS), we further confirmed that *RcERF099* plays a significant role in *B. cinerea* resistance in rose flowers.

## Results

### Identifying *RcERF* genes in the rose genome

In order to identify the potential *AP2/ERFs* of *R. chinensis*, we downloaded the AP2/ERF HMM profile (PF00847) from the Pfam database. Using this profile as a query, the HMM search of the rose genome finally lead to the identification of 137 candidate *RcERF* genes. Conserved Domains Database (https://www.ncbi.nlm.nih.gov/Structure/cdd/wrpsb.cgi) and ExPASy (http://web.expasy.org/protparam/) were employed to verify all candidate *RcERFs* contain a single AP2/ERF motif. We further removed any sequence having less than 150 amino acids, and finally obtained a total of 131 non-redundant *RcERF* genes. All these 131 *ERF* family genes can be mapped onto rose chromosomes and we designated the genes *RcERF001* to *RcERF131* in accordance with their chromosome order.

The length of proteins encoded by *RcERF* family genes varies from 150 to 832 amino acids, with an average length of 298 amino acids. The longest (RcERF052) contains 832 amino acids, whereas the shortest just has 150 amino acids (RcERF093 and RcERF095). Table 1 summarizes detailed information of all 131 *RcERF* genes, including their accession numbers, chromosome locations, exon and intron details, protein size and classification.

**Table 1:** Members of the AP2/ERF gene family in rose genome

Gene	Accession number^**a**^	Chr.^**b**^	Position^**c**^	Intro	Exon	CDS (bp)	AA^**d**^	Subfamily
RcERF001	RchiOBHm_Chr1g0331141	1	20.92	6	7	1203	401	AP2
RcERF002	RchiOBHm_Chr1g0346421	1	38.78	0	1	831	277	DREB
RcERF003	RchiOBHm_Chr1g0347621	1	40.31	0	1	819	273	ERF
RcERF004	RchiOBHm_Chr1g0347631	1	40.33	0	1	639	213	ERF
RcERF005	RchiOBHm_Chr1g0347641	1	40.38	0	1	717	239	ERF
RcERF006	RchiOBHm_Chr1g0347661	1	40.38	0	1	651	217	ERF
RcERF007	RchiOBHm_Chr1g0347671	1	40.38	0	1	612	204	ERF
RcERF008	RchiOBHm_Chr1g0349631	1	42.73	0	1	711	237	ERF
RcERF009	RchiOBHm_Chr1g0358681	1	50.76	0	1	903	301	ERF
RcERF010	RchiOBHm_Chr1g0360021	1	51.85	0	1	633	211	DREB
RcERF011	RchiOBHm_Chr1g0360081	1	51.90	2	3	1032	344	DREB
RcERF012	RchiOBHm_Chr1g0364341	1	55.52	8	9	1371	457	AP2
RcERF013	RchiOBHm_Chr1g0370631	1	60.12	0	1	987	329	DREB
RcERF014	RchiOBHm_Chr1g0371151	1	60.47	1	1	1152	384	DREB
RcERF015	RchiOBHm_Chr1g0373621	1	61.76	0	1	858	286	ERF
RcERF016	RchiOBHm_Chr1g0373631	1	61.77	0	1	879	293	ERF
RcERF017	RchiOBHm_Chr1g0373641	1	61.77	0	1	642	214	ERF
RcERF018	RchiOBHm_Chr1g0376641	1	63.85	0	1	693	231	DREB
RcERF019	RchiOBHm_Chr1g0376651	1	63.86	0	1	699	233	DREB
RcERF020	RchiOBHm_Chr1g0380021	1	65.82	0	1	1092	364	ERF
RcERF021	RchiOBHm_Chr2g0088321	2	2.93	1	2	615	205	DREB
RcERF022	RchiOBHm_Chr2g0091471	2	5.12	0	1	765	255	DREB
RcERF023	RchiOBHm_Chr2g0095581	2	8.53	0	1	630	210	DREB
RcERF024	RchiOBHm_Chr2g0105221	2	16.56	0	1	699	233	ERF
RcERF025	RchiOBHm_Chr2g0105401	2	16.68	0	1	726	242	ERF
RcERF026	RchiOBHm_Chr2g0105461	2	16.74	0	1	639	213	ERF
RcERF027	RchiOBHm_Chr2g0105481	2	16.76	0	1	579	193	ERF
RcERF028	RchiOBHm_Chr2g0105501	2	16.78	0	1	543	181	ERF
RcERF029	RchiOBHm_Chr2g0105521	2	16.81	0	1	624	208	ERF
RcERF030	RchiOBHm_Chr2g0106221	2	17.67	9	10	1605	535	AP2
RcERF031	RchiOBHm_Chr2g0106241	2	17.71	0	1	519	173	DREB
RcERF032	RchiOBHm_Chr2g0108831	2	20.29	8	9	1980	660	AP2
RcERF033	RchiOBHm_Chr2g0111031	2	22.67	8	8	1629	543	AP2
RcERF034	RchiOBHm_Chr2g0115041	2	27.01	1	1	1047	349	ERF
RcERF035	RchiOBHm_Chr2g0118211	2	30.54	1	2	966	322	ERF
RcERF036	RchiOBHm_Chr2g0118251	2	30.58	1	2	1164	388	ERF
RcERF037	RchiOBHm_Chr2g0126301	2	40.60	0	1	1398	466	ERF
RcERF038	RchiOBHm_Chr2g0130611	2	46.70	0	1	537	179	ERF
RcERF039	RchiOBHm_Chr2g0132251	2	48.70	6	7	1074	358	AP2
RcERF040	RchiOBHm_Chr2g0133451	2	50.24	1	2	603	201	DREB
RcERF041	RchiOBHm_Chr2g0133601	2	50.47	0	1	888	296	ERF
RcERF042	RchiOBHm_Chr2g0135921	2	53.15	1	2	582	194	DREB
RcERF043	RchiOBHm_Chr2g0139661	2	57.18	0	1	786	262	DREB
RcERF044	RchiOBHm_Chr2g0145271	2	62.91	8	9	1731	577	AP2
RcERF045	RchiOBHm_Chr2g0147651	2	65.22	2	2	1176	392	ERF
RcERF046	RchiOBHm_Chr2g0157901	2	74.24	0	1	693	231	ERF
RcERF047	RchiOBHm_Chr2g0160621	2	76.47	1	1	582	194	DREB
RcERF048	RchiOBHm_Chr2g0163201	2	78.78	0	1	909	303	RAV
RcERF049	RchiOBHm_Chr2g0166851	2	81.58	0	1	1071	357	ERF
RcERF050	RchiOBHm_Chr2g0167081	2	81.74	0	1	1257	419	ERF
RcERF051	RchiOBHm_Chr2g0169071	2	83.36	4	5	1377	459	AP2
RcERF052	RchiOBHm_Chr3g0447531	3	0.21	7	8	2496	832	AP2
RcERF053	RchiOBHm_Chr3g0449251	3	1.12	9	8	804	268	Soloist
RcERF054	RchiOBHm_Chr3g0450011	3	1.66	0	1	702	234	ERF
RcERF055	RchiOBHm_Chr3g0450351	3	1.92	0	1	900	300	ERF
RcERF056	RchiOBHm_Chr3g0461691	3	9.68	1	2	1791	597	DREB
RcERF057	RchiOBHm_Chr3g0468481	3	14.49	8	9	1026	342	AP2
RcERF058	RchiOBHm_Chr3g0472281	3	18.19	0	1	615	205	DREB
RcERF059	RchiOBHm_Chr3g0472361	3	18.24	0	1	600	200	DREB
RcERF060	RchiOBHm_Chr3g0480891	3	26.82	5	6	1212	404	AP2
RcERF061	RchiOBHm_Chr3g0481251	3	27.33	0	1	1047	349	DREB
RcERF062	RchiOBHm_Chr3g0482661	3	28.70	8	9	1275	425	AP2
RcERF063	RchiOBHm_Chr4g0392461	4	7.95	0	1	468	156	ERF
RcERF064	RchiOBHm_Chr4g0392501	4	7.98	0	1	804	268	ERF
RcERF065	RchiOBHm_Chr4g0401791	4	20.05	0	1	918	306	ERF
RcERF066	RchiOBHm_Chr4g0401801	4	20.08	8	9	1659	553	AP2
RcERF067	RchiOBHm_Chr4g0405371	4	25.78	6	7	1098	366	AP2
RcERF068	RchiOBHm_Chr4g0415231	4	39.84	0	1	1206	402	ERF
RcERF069	RchiOBHm_Chr4g0421551	4	47.20	1	2	1209	403	ERF
RcERF070	RchiOBHm_Chr4g0423581	4	49.24	1	2	765	255	ERF
RcERF071	RchiOBHm_Chr4g0428551	4	53.58	0	1	813	271	ERF
RcERF072	RchiOBHm_Chr4g0428891	4	53.79	1	2	708	236	ERF
RcERF073	RchiOBHm_Chr4g0433071	4	57.25	0	1	1284	428	ERF
RcERF074	RchiOBHm_Chr4g0435261	4	58.89	1	1	1041	347	DREB
RcERF075	RchiOBHm_Chr4g0435771	4	59.21	0	1	1098	366	RAV
RcERF076	RchiOBHm_Chr4g0440541	4	62.65	5	6	1299	433	AP2
RcERF077	RchiOBHm_Chr5g0008991	5	5.94	0	1	792	264	ERF
RcERF078	RchiOBHm_Chr5g0009711	5	6.43	0	1	510	170	ERF
RcERF079	RchiOBHm_Chr5g0009741	5	6.45	0	1	804	268	ERF
RcERF080	RchiOBHm_Chr5g0032721	5	26.47	0	1	750	250	ERF
RcERF081	RchiOBHm_Chr5g0041261	5	36.01	0	1	678	226	ERF
RcERF082	RchiOBHm_Chr5g0046591	5	42.67	0	1	1098	366	RAV
RcERF083	RchiOBHm_Chr5g0061501	5	67.00	5	6	855	285	AP2
RcERF084	RchiOBHm_Chr5g0073531	5	79.54	0	1	798	266	ERF
RcERF085	RchiOBHm_Chr5g0077201	5	83.01	7	8	1659	553	AP2
RcERF086	RchiOBHm_Chr5g0080541	5	86.52	0	1	1095	365	RAV
RcERF087	RchiOBHm_Chr5g0083271	5	88.95	0	1	846	282	ERF
RcERF088	RchiOBHm_Chr6g0257181	6	12.45	0	1	804	268	ERF
RcERF089	RchiOBHm_Chr6g0274591	6	36.05	1	2	1353	451	ERF
RcERF090	RchiOBHm_Chr6g0276671	6	38.87	0	1	969	323	ERF
RcERF091	RchiOBHm_Chr6g0284081	6	47.38	6	6	669	223	Soloist
RcERF092	RchiOBHm_Chr6g0288231	6	51.49	0	1	789	263	ERF
RcERF093	RchiOBHm_Chr6g0288241	6	51.53	0	1	450	150	ERF
RcERF094	RchiOBHm_Chr6g0288261	6	51.55	0	1	522	174	ERF
RcERF095	RchiOBHm_Chr6g0288271	6	51.55	0	1	450	150	ERF
RcERF096	RchiOBHm_Chr6g0288281	6	51.55	0	1	477	159	ERF
RcERF097	RchiOBHm_Chr6g0289271	6	52.38	0	1	636	212	ERF
RcERF098	RchiOBHm_Chr6g0294441	6	56.77	1	2	927	309	ERF
RcERF099	RchiOBHm_Chr6g0295481	6	57.48	0	1	702	234	DREB
RcERF100	RchiOBHm_Chr6g0298011	6	59.58	1	2	684	228	DREB
RcERF101	RchiOBHm_Chr6g0299771	6	60.81	1	2	618	206	DREB
RcERF102	RchiOBHm_Chr6g0301981	6	62.18	0	1	771	257	DREB
RcERF103	RchiOBHm_Chr6g0306191	6	64.95	0	1	747	249	DREB
RcERF104	RchiOBHm_Chr6g0308371	6	66.49	1	1	468	156	DREB
RcERF105	RchiOBHm_Chr6g0310091	6	67.50	8	9	1971	657	AP2
RcERF106	RchiOBHm_Chr7g0184251	7	4.91	0	1	642	214	ERF
RcERF107	RchiOBHm_Chr7g0185311	7	5.49	3	2	1143	381	DREB
RcERF108	RchiOBHm_Chr7g0187951	7	7.65	0	1	975	325	ERF
RcERF109	RchiOBHm_Chr7g0188681	7	8.08	1	2	798	266	ERF
RcERF110	RchiOBHm_Chr7g0188691	7	8.09	1	2	711	237	ERF
RcERF111	RchiOBHm_Chr7g0195031	7	13.00	0	1	561	187	ERF
RcERF112	RchiOBHm_Chr7g0195581	7	13.38	0	1	1005	335	ERF
RcERF113	RchiOBHm_Chr7g0195661	7	13.46	12	9	1464	488	Soloist
RcERF114	RchiOBHm_Chr7g0199231	7	17.30	0	1	840	280	DREB
RcERF115	RchiOBHm_Chr7g0199251	7	17.32	0	1	723	241	DREB
RcERF116	RchiOBHm_Chr7g0199301	7	17.34	0	1	720	240	DREB
RcERF117	RchiOBHm_Chr7g0199331	7	17.37	0	1	723	241	DREB
RcERF118	RchiOBHm_Chr7g0199351	7	17.38	0	1	753	251	DREB
RcERF119	RchiOBHm_Chr7g0199381	7	17.42	0	1	726	242	DREB
RcERF120	RchiOBHm_Chr7g0203971	7	21.55	0	1	669	223	DREB
RcERF121	RchiOBHm_Chr7g0204031	7	21.62	0	1	537	179	DREB
RcERF122	RchiOBHm_Chr7g0204611	7	22.29	0	1	1023	341	ERF
RcERF123	RchiOBHm_Chr7g0204641	7	22.33	1	2	876	292	ERF
RcERF124	RchiOBHm_Chr7g0230931	7	54.58	1	2	561	187	DREB
RcERF125	RchiOBHm_Chr7g0231481	7	55.10	0	1	498	166	DREB
RcERF126	RchiOBHm_Chr7g0231501	7	55.11	0	1	498	166	DREB
RcERF127	RchiOBHm_Chr7g0231631	7	55.25	0	1	588	196	DREB
RcERF128	RchiOBHm_Chr7g0231641	7	55.30	0	1	582	194	DREB
RcERF129	RchiOBHm_Chr7g0231921	7	55.76	0	1	582	194	DREB
RcERF130	RchiOBHm_Chr7g0235201	7	59.94	0	1	552	184	DREB
RcERF131	RchiOBHm_Chr7g0239701	7	65.48	0	1	1131	377	ERF

^a^Available at https://lipm-browsers.toulouse.inra.fr/pub/RchiOBHm-V2/

^b^Chromosome

^c^Starting position (Mb)

^d^Amino Acids

### Chromosomal localization and microsynteny analysis

**Fig. 1:**
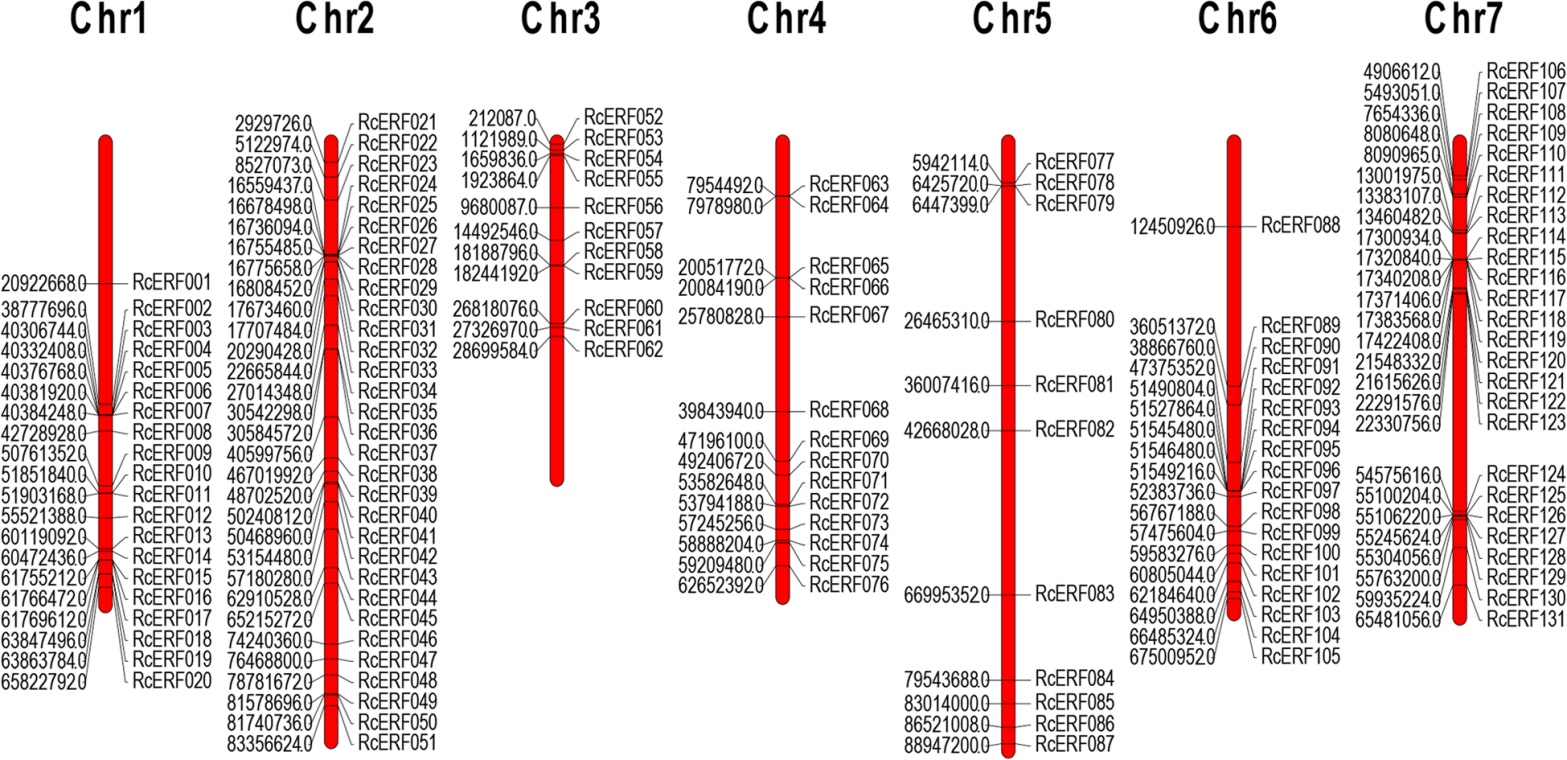
Chromosome localization of rose AP2/ERF family members. The physical distribution of each *RcERF* gene is listed on the seven chromosomes of *Rose chinensis*

131 *RcERF* genes were located on all 7 rose chromosomes, as depicted in Fig. 1. Chromosome 2 contains the largest number of *RcERF* genes (31), followed by chromosome 7 (26). Chromosomes 3 and 5 contain the least number of chromosomes (11). The *RcERF* genes were unevenly distributed over 7 chromosomes. 8.40% of *RcERF*s were located in the long arm of chromosomes 3 and 5, 23.66% of *RcERF*s were located in chromosome 2, 15.27% of *RcERF*s were located in chromosome 1, 10.69 and 13.74% of *RcERF*s were distributed over chromosome 4 and 6. Chromosome 7 contains 19.85% *RcERFs*, and they were distributed over both the long and short arms.

**Fig. 2:**
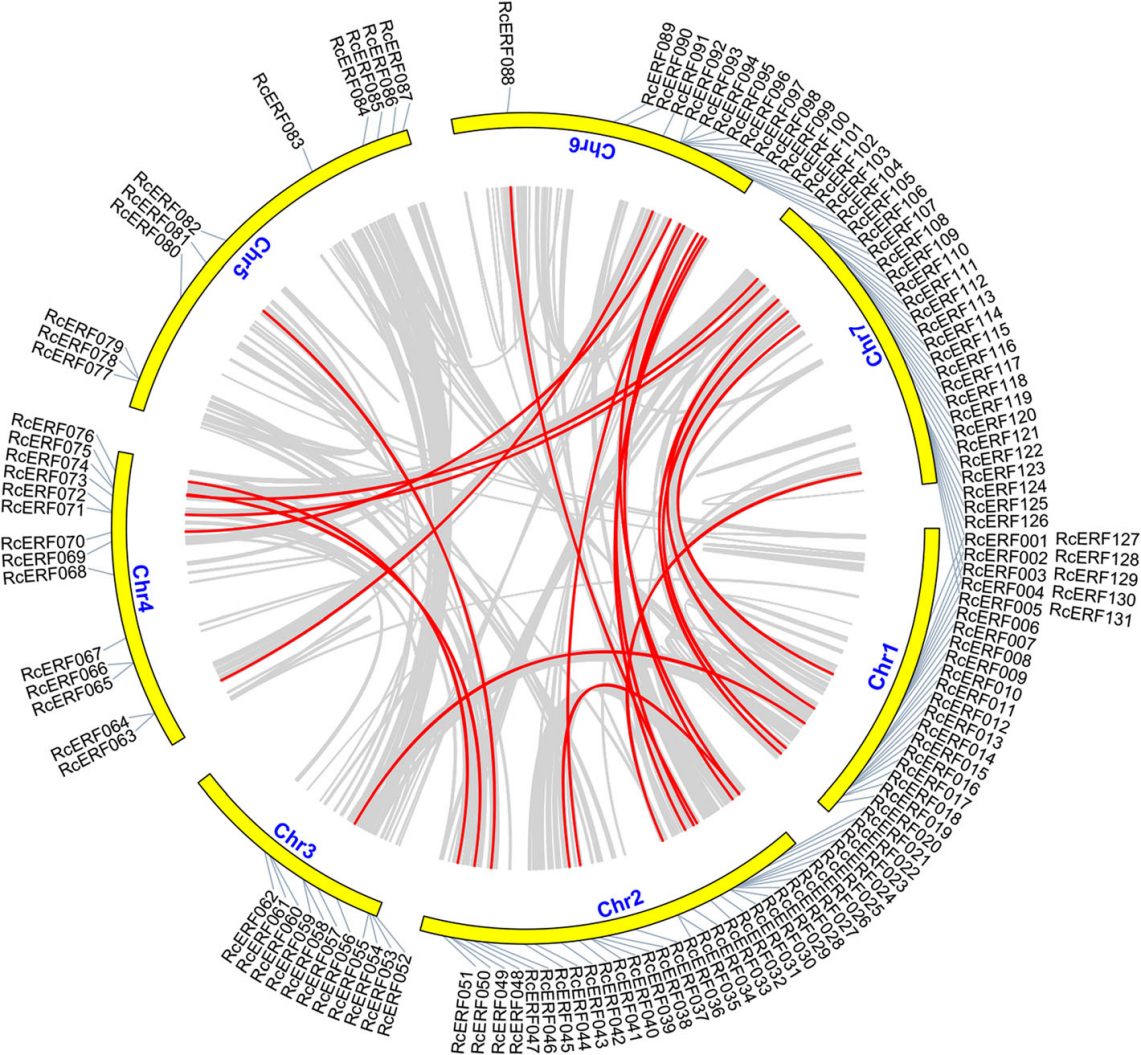
Microsyntenic analyses of the rose AP2/ERF transcription factors in the *Rose chinensis* genome. Circular visualization of rose AP2/ERF transcription factors is mapped onto different chromosomes using Circos. The red lines indicate rose *AP2/ERF* genes having a syntenic relationship. The grey lines represent all syntenic blocks in the genome of *R. chinensis*

Furthermore, we studied *RcERFs* duplication events, and discovered in total 21 gene pairs in the rose genome (Table 2). Only one gene pair was located on the same chromosome (*RcERF021* and *RcERF042*), indicating that they are likely to be tandem repeats. The remaining 20 gene pairs were located on different chromosomes, and indicated that segmental duplication may occur in these regions (Fig. 2).

**Table 2:** Duplication analysis of the AP2/ERF gene family

Sequence 1	Sequence2	Ka	Ks	Ka_Ks	Effective Len	Average S-sites	Average N-sites
RcERF021	RcERF042	0.29553678	1.72567726	0.1712584	582	132	450
RcERF012	RcERF057	0.40300562	1.38085301	0.2918527	924	212.75	711.25
RcERF048	RcERF075	0.4114621	NaN	NaN	900	197.4166667	702.5833333
RcERF051	RcERF076	0.33331089	2.56556843	0.129917	1209	275.3333333	933.6666667
RcERF046	RcERF081	0.3163392	1.85921206	0.1701469	609	153.4166667	455.5833333
RcERF025	RcERF088	0.57783254	1.78941311	0.3229174	708	160.9166667	547.0833333
RcERF064	RcERF092	0.35723109	NaN	NaN	699	158	541
RcERF063	RcERF093	0.36996467	1.47353077	0.2510736	432	104.4166667	327.5833333
RcERF070	RcERF098	0.6685266	1.81097809	0.3691522	753	174	579
RcERF021	RcERF100	0.38250295	1.50870683	0.2535303	612	138.9166667	473.0833333
RcERF040	RcERF101	0.27568714	NaN	NaN	561	126.0833333	434.9166667
RcERF022	RcERF103	0.41399228	1.28764002	0.3215124	735	178.9166667	556.0833333
RcERF031	RcERF104	0.27070983	1.29444056	0.2091327	429	104.0833333	324.9166667
RcERF032	RcERF105	0.27018563	1.27442854	0.2120053	1797	397.1666667	1399.833333
RcERF074	RcERF107	0.76307193	NaN	NaN	969	216.1666667	752.8333333
RcERF072	RcERF109	0.57052476	1.55144847	0.3677368	684	155.4166667	528.5833333
RcERF009	RcERF112	0.56506363	2.56420719	0.2203658	852	194.25	657.75
RcERF020	RcERF112	0.48408323	NaN	NaN	972	229.5	742.5
RcERF019	RcERF119	0.62960209	2.53219954	0.2486384	666	161.75	504.25
RcERF003	RcERF123	0.5452034	2.76643897	0.1970777	759	188.8333333	570.1666667
RcERF034	RcERF131	0.34870274	1.21479419	0.2870468	1011	238.8333333	772.1666667

To explore the selective constraints among duplicated *RcERF* genes, we calculated the ratio of non-synonymous (Ka) to synonymous (Ks) nucleotide substitutions (Ka/Ks ratio) of 21 pairs of duplicated genes (Table 2). A Ka/Ks ratio < 1 indicates a negative or purifying selection of gene pairs, whereas Ka/Ks > 1 depicts a positive selection. Our study revealed that the Ka/Ks ratio for all *RcERF* gene pairs is < 0.4 (Table 2). These data indicate that *RcERF* gene pairs had undergone a purifying selection, and functional differentiation is limited.

### Phylogenetic and exon-intron structural analysis of *RcERF* genes

We performed a phylogenetic analysis on all *RcERF* genes using the neighbor-joining method and established a phylogenetic tree. According to their evolutionary relationships, *RcERF* genes are further categorized into five subfamilies with supported bootstrap values, including ERF, DREB, AP2, RAV and Soloist, comprising 64, 42, 18, 4 and 3 members, respectively.

**Fig. 3:**
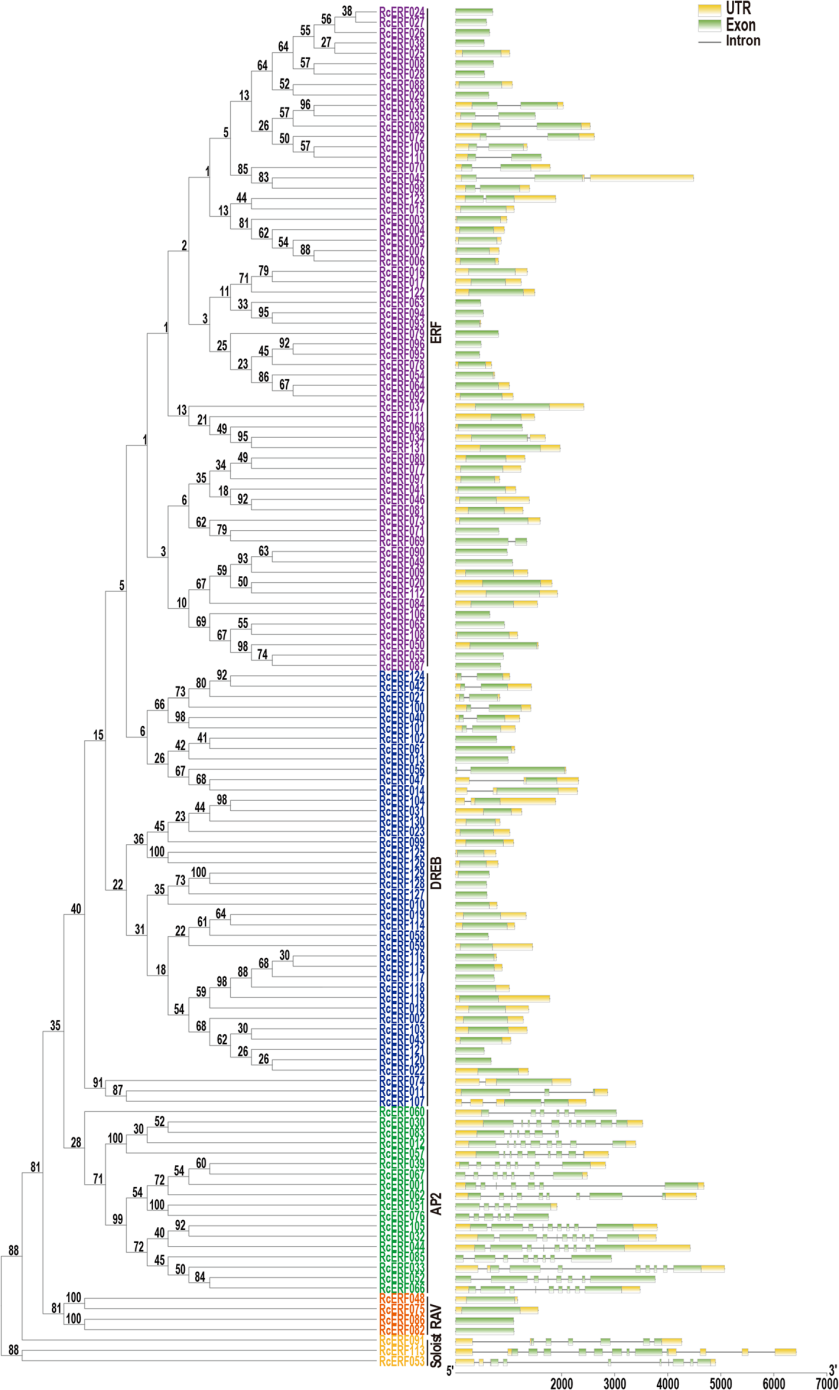
Phylogenetic and gene structural analysis of rose AP2/ERF transcription factors. The phylogenetic tree is constructed by MEGA6.0 using a Neighbor-joining method. Numbers on the nodes of the branches represent bootstrap values. The gene structure diagram represents UTRs, exons and introns with green boxes, yellow boxes and gray lines, respectively. The scale at the bottom estimated the size of UTRs, exons and introns

Subsequent analysis of the exon-intron structure proved to be consistent with the phylogenetic analysis results. Most of the genes clustered in the same subfamily exhibit a similar exon-intron structure. Members of the RAV subfamily do not comprise intron, however, in contrast, AP2 and Soloist subfamily genes comprise four to twelve introns. Most of the ERF and DREB subfamily members have either no intron or only one, however, some exceptions were also observed; for example, *RcERF011* and *RcERF045* have two introns and *RcERF107* has three (Fig. 3; Table 1). These results demonstrate the presence of highly conserved structures within the subfamilies and diversity among the different subfamilies.

**Fig. 4:**
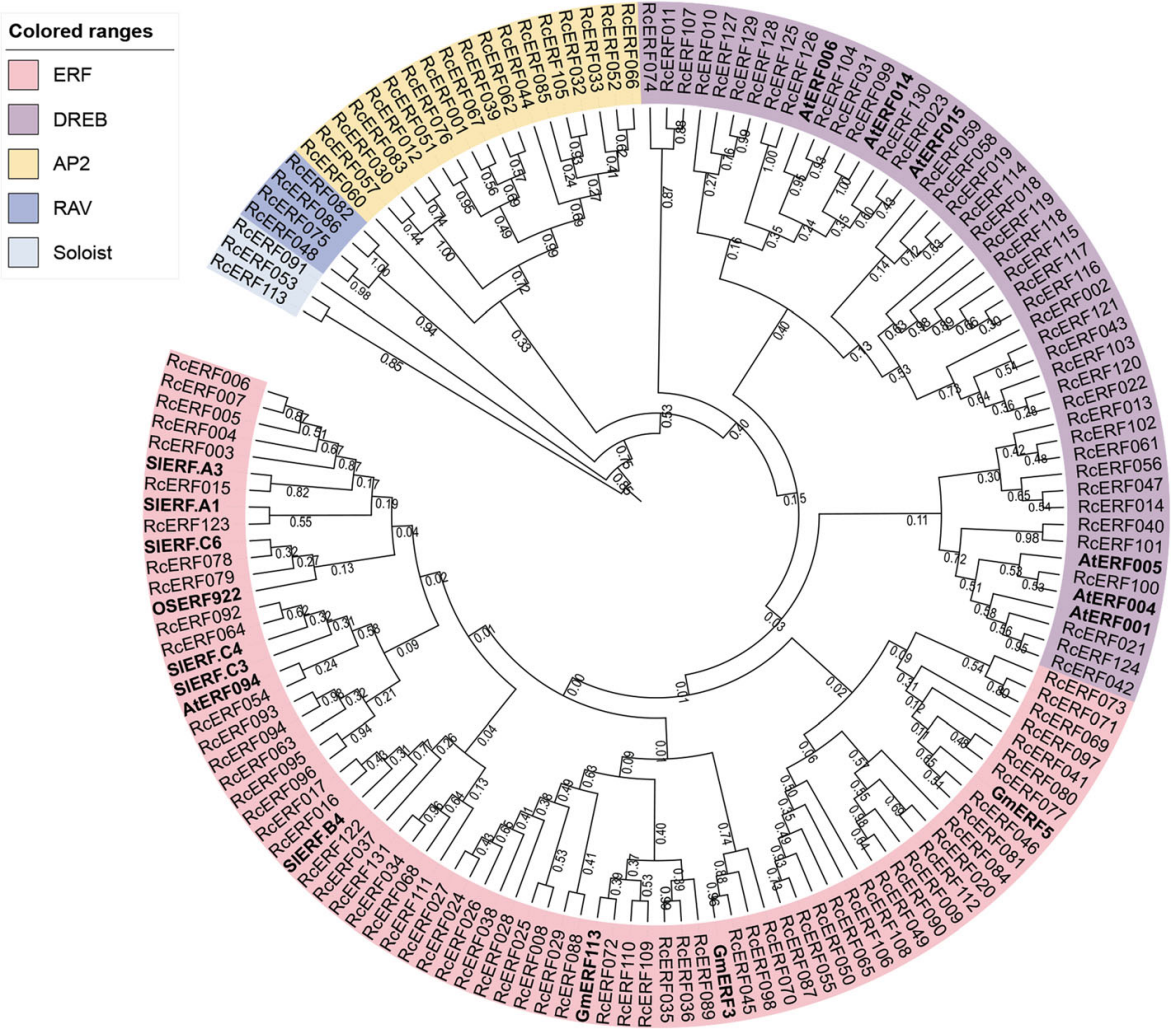
Phylogenetic analyses of the rose AP2/ERF transcription factors with disease-resistance-related AP2/ERF transcription factors from other plant species. The composite phylogenetic tree that included all rose AP2/ERF transcription factors and disease-resistance-related AP2/ERF transcription factors (in bold) from Arabidopsis (*Arabidopsis thaliana*), rice (*Oryza sativa*), soybean (*Glycine max*) and tomato (*Solanum lycopersicum*) were constructed by MEGA 6.0 with the neighbor-Joining method. The bootstrap consensus tree inferred from 1000 replicates is taken to represent the evolutionary history of the taxa analyzed. The bootstrap values are indicated on the nodes of the branches

There is increasing evidence that AP2/ERF transcription factors play a key role in disease resistance in various plant species (Table 3). In order to evaluate *RcERFs*’ involvement in rose disease resistance, we generated a composite phylogenetic tree that included defence-related ERFs in other plant species and all RcERFs (Fig. 4). In this composite phylogenetic tree, each subfamily is marked with a different colour, and all plant ERFs that are known to be involved in disease resistance are in bold. ERFs involved in regulating defence responses are distributed in ERF and DREB subfamilies, but not in AP2, RAV, or Soloist.

**Table 3:** Plant AP2/ERF family genes involved in disease resistance

Gene name	Gene ID	Species	Pathogens	References
*OSERF922*	Os01g54890.1	*Oryza sativa* L.	*Magnaporthe oryzae*	[17]
*GmERF3*	ACD47129.1	*Glycine max*	disease resistance	[18]
*GmERF113*	XP_003548854.1	Glycine max	*Phytophthora sojae*	[19]
*GmERF5*	AEX25891.1	Glycine max	*Phytophthora sojae*	[20]
*AtERF15*	At4g31060	*Arabidopsis thaliana*	*B.cinerea* and DC3000	[21]
*AtERF14*	At1g04370	Arabidopsis thaliana	*Fusarium oxysporum*	[22]
*AtERF1*	At3g2340	Arabidopsis thaliana	*B.cinerea*	[23]
*AtERF5*	At5g47230	Arabidopsis thaliana	*B.cinerea*	[14]
*AtERF4*	At3g15210	Arabidopsis thaliana	Plant defense systems	[7]
*AtERF6*	At4g17490	Arabidopsis thaliana	*B.cinerea*	[14]
*AtERF094(ORA59)*	At1g06160	Arabidopsis thaliana	plant defense	[24]
*SlERF.A1*	Solyc08g078180.1	*Solanum lycopersicum*	*B.cinerea*	[12]
*SlERF.B4*	Solyc03g093540	Solanum lycopersicum	*B.cinerea*	[12]
*SlERF.C3*	Solyc09g066360	Solanum lycopersicum	*B.cinerea*	[12]
*SlERF.A3*	Solyc05g052050	Solanum lycopersicum	*B.cinerea*	[12]
*SlERF.C6*	Solyc02g077370	Solanum lycopersicum	*Pseudomonassyringae* to *pv*.	[25]
*SlERF.C4*	Solyc09g089930	Solanum lycopersicum	Ralstonia Solanacearum Strain BJ1057	[26]

### The expression of *RcERF* genes in response to *Botrytis cinerea* infection

There has been an increasing rise in evidence gained from studying various plant species which indicates that plant AP2/ERF transcription factors play a significant role in pathogen response. In order to study the role of *RcERFs* in *B. cinerea* resistance, we analyzed transcriptome data in rose petals at 30 hpi and 48 hpi of this pathogen. The 30 hpi timepoint represents the early response to infection, whereas the 48 hpi timepoint corresponds to the late response [16]. A total of 23 *RcERF* genes (*RhERF004, RhERF005, RhERF015, RhERF019, RhERF023, RhERF024, RhERF054, RhERF063, RhERF064, RhERF066, RhERF068, RhERF070, RhERF072, RhERF080, RhERF088, RhERF089, RhERF092, RhERF093, RhERF095, RhERF099, RhERF114, RhERF123 and RhERF125*) were significantly up-regulated, indicating they could be key regulators in resisting *B. cinerea* infection in rose. Amongst these *B. cinerea*-induced *RcERFs*, the expression of 10 *RcERF* genes was increased significantly at 30 hpi, suggesting that these *RcERFs* may well be involved in an early response to *B. cinerea* (Table 4).

**Table 4:** Expression of the Rose AP2/ERF genes under *B. cinerea* infection^a^

Gene^**b**^	Accession number	Subfamily	log_**2**_Ratio 30hpi	log_**2**_Ratio 48hpi
RcERF004	RchiOBHm_Chr1g0347631	ERF	–	14.996
RcERF005	RchiOBHm_Chr1g0347641	ERF	–	5.460
RcERF015	RchiOBHm_Chr1g0373621	ERF	1.582	2.148
**RcERF019**	RchiOBHm_Chr1g0376651	DREB	–	2.259
RcERF023	RchiOBHm_Chr2g0095581	DREB	2.100	5.019
RcERF024	RchiOBHm_Chr2g0105221	ERF	–	16.346
RcERF054	RchiOBHm_Chr3g0450011	ERF	–	8.381
**RcERF063**	RchiOBHm_Chr4g0392461	ERF	–	8.895
**RcERF064**	RchiOBHm_Chr4g0392501	ERF	4.876	6.106
RcERF066	RchiOBHm_Chr4g0401801	AP2	–	14.732
RcERF068	RchiOBHm_Chr4g0415231	ERF	–	5.509
**RcERF070**	RchiOBHm_Chr4g0423581	ERF	2.100	3.775
**RcERF072**	RchiOBHm_Chr4g0428891	ERF	1.087	1.803
RcERF080	RchiOBHm_Chr5g0032721	ERF	2.367	2.197
**RcERF088**	RchiOBHm_Chr6g0257181	ERF	–	3.241
RcERF089	RchiOBHm_Chr6g0274591	ERF	1.206	2.469
**RcERF092**	RchiOBHm_Chr6g0288231	ERF	6.085	6.755
**RcERF093**	RchiOBHm_Chr6g0288241	ERF	3.650	6.087
RcERF095	RchiOBHm_Chr6g0288271	ERF	–	7.574
RcERF099	RchiOBHm_Chr6g0295481	DREB	–	4.523
RcERF114	RchiOBHm_Chr7g0199231	DREB	–	3.194
**RcERF123**	RchiOBHm_Chr7g0204641	ERF	1.837	2.980
RcERF125	RchiOBHm_Chr7g0231481	DREB	–	5.621

^a^The log2 transformed expression profiles were obtained from the RNA-seq dataset [16]

^b^The *RcERFs* undergo duplicate events are marked in bold

**Fig. 5:**
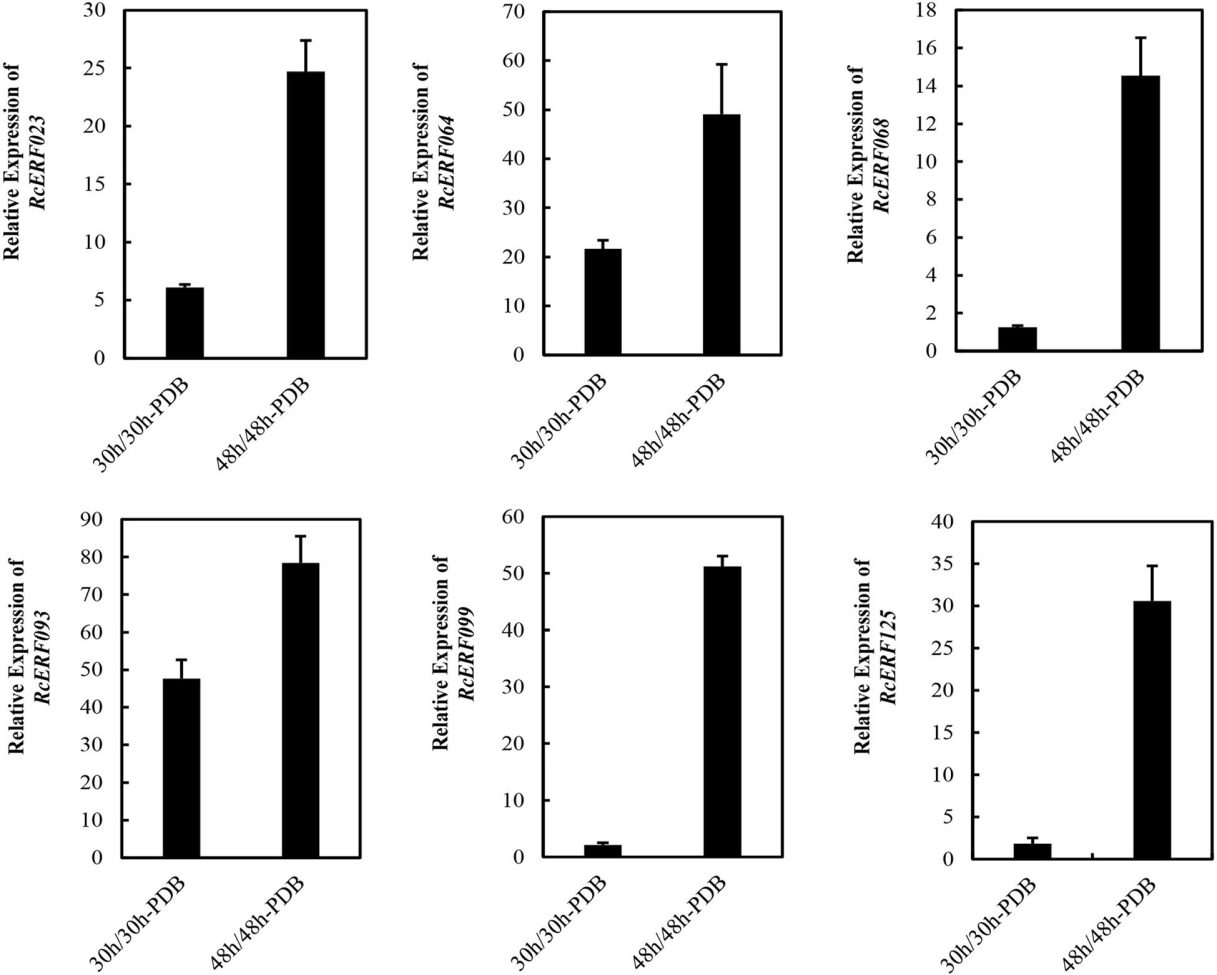
Validation of RNA-Seq results using qPCR. *RcUBI2* was used as a housekeeping gene. Expression profile data of six *RcERF* genes at 30 hpi and 48 hpi after *B. cinerea* inoculation were obtained using qPCR. Error bar represent SD in three technical replicates. The primers used are listed in Supplementary Table S1

In order to further verify the expression profile from RNA-seq, the expression of six *RcERF*s was analyzed by qPCR. The results of the qPCR analysis proved to be consistent with the expression profile obtained from the transcriptome analysis (Fig. 5).

### RcRF099 is required for rose resistance to *B. cinerea*

In order to further illustrate the potential role of *B. cinerea*-induced *RcERF* genes in resistance of this pathogen, we used VIGS to knock down the expression of *RcERF099* in rose petals. *RcERF099* was selected to conduct this VIGS study because: 1) *RcERF099* is up-regulated upon *B. cinerea* infection (Fig. 5; Table 4); and 2) based on phylogenetic analysis, *RcERF099* belongs to the DREB subfamily which comprises many disease-resistant *ERFs* originating from other plant species, such as *AtERF001*, *AtERF004*, *AtERF005*, *AtERF006*, *AtERF014*, and *AtERF015* (Fig. 4; Table 3).

**Fig. 6:**
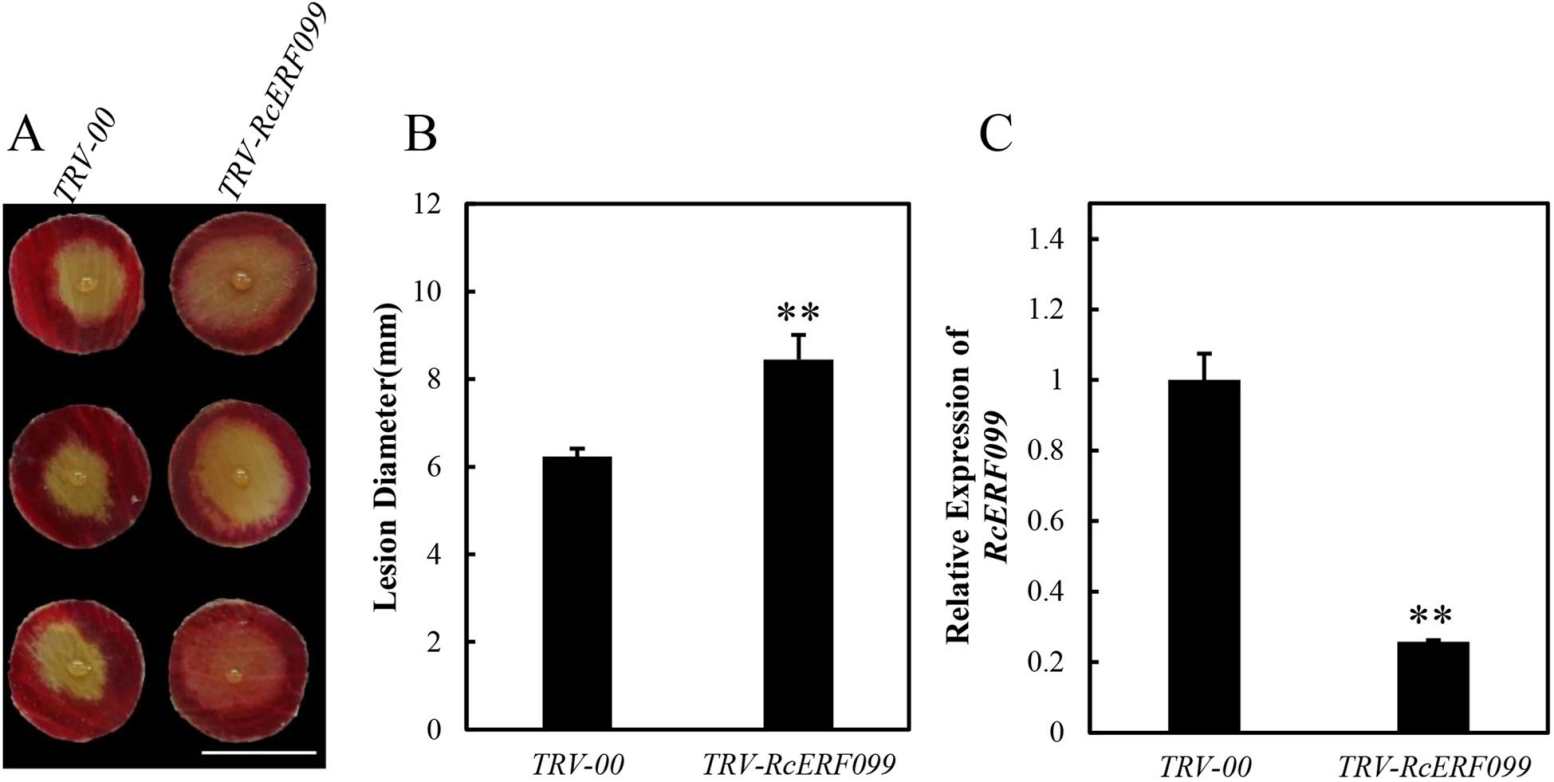
Functional analysis of rose AP2/ERF transcription factor gene *RcERF099*. **a** Compromised *B. cinerea* resistance upon silencing of *RcERF099* (*TRV- RcERF099*) was observed at 60 hpi post-inoculation. **b**. Quantification of *B. cinerea* disease lesions on TRV-RcERF099- and TRV-00-inoculated rose petal discs. The graph indicates the lesion size of three biological replicates (*n* = 48) with the standard deviation. **c**. Expression of *RcERF099* relative to that during the control at 6 days of post-silencing. All statistical analyses were performed using Student’s *t*-test; ** *p* < 0.01

In order to silence *RcERF099* in rose petals, we cloned a 230 bp fragment of *RcERF099* into a pTRV2 vector [27] to generate *TRV-RcERF099*. *Agrobacterium tumefaciens* carrying *TRV-RcERF099* and *TRV1* [27] were co-infiltrated into rose petal discs to generate *RcERF099*-silenced rose petals. The infiltrated rose petal discs were then inoculated with *B. cinerea*. Comparing the control petal (*TRV-00*) inoculated with an empty TRV, the plant inoculated with *TRV-RcERF099* showed more serious disease symptoms displaying a significant increase in the size of the disease lesion (Fig. 6a and b). Furthermore, we confirmed the silencing efficiency of VIGS with qPCR (Fig. 6c). These results indicated that *RcERF099* is required for rose resistance to *B. cinerea*.

## Discussion

Plant disease resistance-related genes are often induced by the invasion of pathogens, and are regulated at the transcriptional level by specific transcription factors. The AP2/ERFs is a major transcription factor family in plants, and has proved to have important functions in disease resistance in various plant species [28–32]. A genome-wide analysis of the AP2/ERF gene family has been performed in arabidopsis and rice [4]. So far, no comprehensive analysis of the rose AP2/ERF gene family has yet been reported, and the function of most *RcERFs* is largely generally unknown. In the current study, using the recently available rose genome, we performed a comprehensive analysis of the AP2/ERF gene family, including their gene structure, phylogeny, chromosomal location, gene duplication, as well as expression profiles during infection of gray mold caused by necrotrophic fungal pathogen *B. cinerea*.

The number of *AP2/ERF* genes in rose (131) has proved to be lower than those in arabidopsis (147) and rice (164) [4], which indicates that the AP2/ERF gene family in different plants has expanded in various degrees during its evolution. Furthermore, we indicated that gene duplication is involved in the expansion of the *RcERF* gene family, in which a total of 21 duplication events were identified. Most of the duplicated genes (20) were involved in segmental duplication, whereas only one was involved in tandem duplication. Interestingly, the Ka/Ks ratio of all these 21 *RcERF* duplicates was < 1, indicating that the *RcERF* gene family undergoes a purification rather than a positive selection, suggesting a highly conservative evolution of this important transcription factor in the gene family. Previously, it has been demonstrated that the plant immune receptor genes involved in race-specific recognition of an invading pathogen undergo positive selection pressure [15]. It further indicates that the *RcERFs* generally involved in the basal defence against pathogens, are not race-specific resistance.

Although the role of *RcERFs* in disease resistance remains unclear, increasing evidence has proved that plant *AP2/ERF* genes are important players involved in regulating plant disease resistance. It prompts us to search for candidate *RcERFs* that are involved in the resistance to *B. cinerea* in roses. Based on their expression in response to gray mold infestation, we identified 23 *RcERFs* that could well be involved in gray mold resistance in rose petals.

We subsequently added plant ERFs that are known to be involved in disease resistance in the RcERFs phylogenetic tree. We discovered that these disease-related ERFs are mainly distributed within ERF and DREB subfamilies. The *RcERF099* belongs to the DREB subfamily, which includes certain members of known disease-related plant *ERF* genes (Fig. 4). Especially, RcERF099 has a close homolog with Arabidopsis AtERF014, which has proved to play an important role in resistance against both bacterial pathogen *Pseudomonas syringae* pv. tomato, as well as fungal pathogen *Fusarium oxysporum* and *B. cinerea* [22]. More importantly, *RcERF099* was induced significantly with *B. cinerea*. We therefore consider that *RcERF099* should be regarded as an important candidate gene involved in the regulation of rose disease resistance. The silencing of *RcERF099* in rose petals by VIGS increased its susceptibility to *B. cinerea*, indicating that it has a positive regulatory function in gray mold resistance.

## Conclusion

pt?>In this study, a genome-wide analysis of *RcERFs* was carried out. A total of 131 non-redundant *AP2/ERF* family members were identified in the rose genome, and these *RcERFs* were divided into 5 subfamilies on the basis of phylogeny and conserved domains. Expression analysis indicated that the transcriptional regulation of certain *RcERF* family genes was induced by *B. cinerea* infection in rose petals. In addition, plant ERFs involved in disease resistance are usually clustered on the same branch of the phylogenetic tree. Based on these analyses, using VIGS, we further proved that *RcERF099* is involved in regulating resistance to *B. cinerea* in rose petals. The information ensuing from these results may facilitate further research into *RcERFs* functions and crop improvement.

## Methods

### Identification of the rose AP2/ERF family gene

The genome sequences and CDS sequences of rose were downloaded from the website (https://lipm-browsers.toulouse.inra.fr/pub/RchiOBHm-V2/) to construct a local genome database. Based on AP2/ERF HMM (Hidden Markov model) from Pfam (PF00847, http://pfam.xfam.org), we initially identified AP2/ERF candidate genes in the rose genome with E-value <1e^− 3^. Finally, all candidate AP2/ERF sequences were verified that they contain at least one AP2/ERF domain through the CDD (Conserved Domains Database; https://www.ncbi.nlm.nih.gov/Structure/cdd/wrpsb.cgi) and ExPASy (http://web.expasy.org/protparam/). Sequences without relevant domains or conserved motifs were removed. Chromosomal distribution of each *AP2/ERF* gene was mapped using Mapchart 2.2 software [33].

### Gene structure and phylogenetic analysis of RcERFs

The map of exon-intron structures of the *RcERF* genes was carried out using TBtools software [34] by comparing the coding sequences (CDS) with their corresponding protein sequences. Furthermore, the phylogenetic analysis of *RcERFs* in the rose was conducted using the NJ method in MEGA 6.0 software and the bootstrap test was carried out with 1000 replicates.

In addition, 17 *ERFs* were previously reported that involved in disease resistance. These *ERFs* originate from various plant species, including tomato (*Solanum lycopersicum*), rice (*Oryza sativa*), soybean (*Glycine max*), and *Arabidopsis thaliana*. Amino acid sequences of these disease resistance-related ERFs and rose AP2/ERFs were then aligned using ClustalW. The alignment of protein sequences which resulted was subsequently used for phylogenetic analysis. A phylogenetic analysis was conducted using the NJ method in MEGA 6.0 software [35] and the bootstrap test was carried out with 1000 replicates. On the phylogenetic dendrograms, the percentage of replicated trees in which the associated taxa clustered together in the bootstrap test is indicated alongside the branches.

### Collinearity analyses

For the purpose of identifying the collinearity of *RcERFs*, we downloaded the genome sequence of rose on a local server, and a Multiple Collinearity Scan toolkit [36] was used to determine microsyntenic relationships between *RcERF* genes. The resultant microsynteny relationships were further evaluated by CollinearScan set at an E-value of <1e^− 10^.

### Calculation of non-synonymous (Ka) to synonymous (Ks) substitution rates

TBtools was used to calculate the synonymous (Ks) and non-synonymous (Ka) nucleotide substitution rates. The Ka/Ks ratios of duplicated gene pairs were calculated to determine the selection mode driving the evolution of *RcERFs*.

### Expression of *RcERFs* in response to *B. cinerea*

RNA-Seq data (accession number PRJNA414570) of rose petals undergoing *B. cinerea* infection was downloaded from the National Center for Biotechnology Information (NCBI) database. The clean sequencing reads were mapped to the *Rosa chinensis* ‘Old Blush’ reference genome. Gene expression levels of *RcERFs* were calculated by Reads per kb per million reads (RPKM). And differentially expressed gene based on Log2 fold change was performed by DEseq2. In order to verify the RNA-Seq results, the expression of 6 *RcERF* genes was analyzed using quantitative PCR (qPCR). To this end, total RNA was extracted from rose petals at 30 h and 48 h post-inoculation (hpi) respectively with *B. cinerea* using the hot borate method as previously described [37]. One microgram of DNase-treated RNA was used to synthesize the first-strand cDNA by using HiScript II Q Select RT SuperMix (Vazyme) in a 20-μL reaction volume. An qPCR reaction was performed using the SYBR Green Master Mix (Takara), and detection was achieved in StepOnePlus Real-Time PCR System (Thermo Fisher Scientific). *RcUBI2* was used as an internal control. A delta-delta-Ct method calculation method was used for expression analysis. All primers that were used as qPCR are listed in Supplementary Table S1.

### VIGS and *B. cinerea* inoculation assays

The rose plants (*Rosa hybrida*) used in this study were grown in soil in a greenhouse in Yunnan, China. In order to obtain the constructs for silencing, a 230 bp sequence of *RcERF099* was amplified using primers TRV-RcERF099-F (5′- GGGGACAAGTTTGTACAAAAAAGCAGGCTGCTCATTTGGGTCCTATACT − 3′) and TRV-RcERF099-R (5′- GGGGACCACTTTGTACAAGAAAGCTGGGTAGTAATATCTTCAAGCAATT − 3′). The fragment generated was subsequently cloned into *TRV2* vectors [27]. The VIGS of detached rose petal discs has been described previously [38]. In brief, detached petals are obtained from the outermost whorls of the rose, and 15-mm petal discs were punched. Agrobacterium consisting of *TRV1* [27] and *TRV2* constructs were mixed at a ratio of 1: 1 and vacuum infiltrated into petal discs. Petal discs were then inoculated with *B. cinerea* at 6 days after TRV infection. At least three biological repeats were performed, using at least 16 discs for each repeat. The disease lesion was estimated at 60 h post-inoculation, and a Student’s *t*-test conducted to determine the significance. All primers used for this study are listed in Supplementary Table S1.

## Supplementary Information

**Additional file 1: Table S1.** List of primers used in this study.

**Additional file 2: Figure S1.** Melting curves for qPCR.

## Data Availability

The datasets used and/or analyzed during the current study have been included within supplemental data. The Raw data of RNA-Seq of rose petals undergoing *B. cinerea* infection can be found in the BioProject database (accession nr. PRJNA414570). The plant materials are available from the corresponding author on request.

## Abbreviations

hpi: Hours post-inoculation
NJ: Neighbor-joining
HMM: Hidden Markov Model
CDD: Conserved Domains Database
VIGS: Virus-induced gene silencing
