# Supplementary material for: Global analysis of the AP2/ERF gene family in rose (Rosa chinensis) genome unveils the role of RcERF099 in Botrytis resistance
Source: BMC Plant Biol. 2020 Nov 23;20:533. doi: 10.1186/s12870-020-02740-6 (PMC7684944; doi:10.1186/s12870-020-02740-6)
Supplement: Supplementary file 1 — Additional file 1: Table S1. List of primers used in this study. [file 12870_2020_2740_MOESM1_ESM.docx]

**Supplemental Table S1 List of primers used in this study**

| **Gene name** | **Accession number** | **Primer sequence（5’-3’）** | **Amplicon length** | **Ta** | **Tm** | **Amplification efficiency** |
| --- | --- | --- | --- | --- | --- | --- |
| RcERF023 | RchiOBHm_Chr2g0095581 | F： GCCGCCGCAGGTCCCCAGGAGCCAC | 119bp | 60°C | 87.51 | 1.971 |
|  |  | R： TAAAGTCACTAGCGGCACTGTCCAT |  |  |  |  |
| RcERF064 | RchiOBHm_Chr4g0392501 | F： CAATGAAAACGACTCCGAGGAAATG | 119bp | 60°C | 81.69 | 2.005 |
|  |  | R： CCTTTATGGGGTTGGATGAGGCAGC |  |  |  |  |
| RcERF068 | RchiOBHm_Chr4g0415231 | F： GTTTCTGGTAACTCCACTTTGATAA | 185bp | 60°C | 80.79 | 1.660 |
|  |  | R： AGGGTTGTTGGTTCCTGCGAAAGTA |  |  |  |  |
| RcERF093 | RchiOBHm_Chr6g0288241 | F： GGAGGGGGAGAGAGCAGAGAGTAAT | 119bp | 60°C | 84.1 | 1.972 |
|  |  | R： GAATCTCTGCCGCATACTTACCCCA |  |  |  |  |
| RcERF099 | RchiOBHm_Chr6g0295481 | F： TCCTCAGCCAATCTCAACTTCCCAA | 93bp | 60°C | 79.62 | 2.118 |
|  |  | R： TACTCTCTGAATGGACTTAGGGGAC |  |  |  |  |
| RcERF125 | RchiOBHm_Chr7g0231481 | F： ATGAGTTACAGTTCAGAAGACAGCA | 109bp | 60°C | 85.27 | 1.968 |
|  |  | R： GGATTTCCGACACCCACTTGCCCCA |  |  |  |  |
| RcUBI2 | RchiOBHm_Chr1g0359561 | F： GCCCTGGTGCGTTCCCAACTG | 82bp | 60°C | 82.43 | 2.024 |
|  |  | R： CCTGCGTGTCTGTCCGCATTG |  |  |  |  |

Ta: amplification temperature

Tm: melting temperature
