# Supplementary material for: Global analysis of the AP2/ERF gene family in rose (Rosa chinensis) genome unveils the role of RcERF099 in Botrytis resistance
Source: BMC Plant Biol. 2020 Nov 23;20:533. doi: 10.1186/s12870-020-02740-6 (PMC7684944; doi:10.1186/s12870-020-02740-6)
Supplement: Supplementary file 2 — Additional file 2: Figure S1. Melting curves for qPCR. [file 12870_2020_2740_MOESM2_ESM.pdf]

Supplemental Figure S1 Melting curves for each gene and control :RcERF023

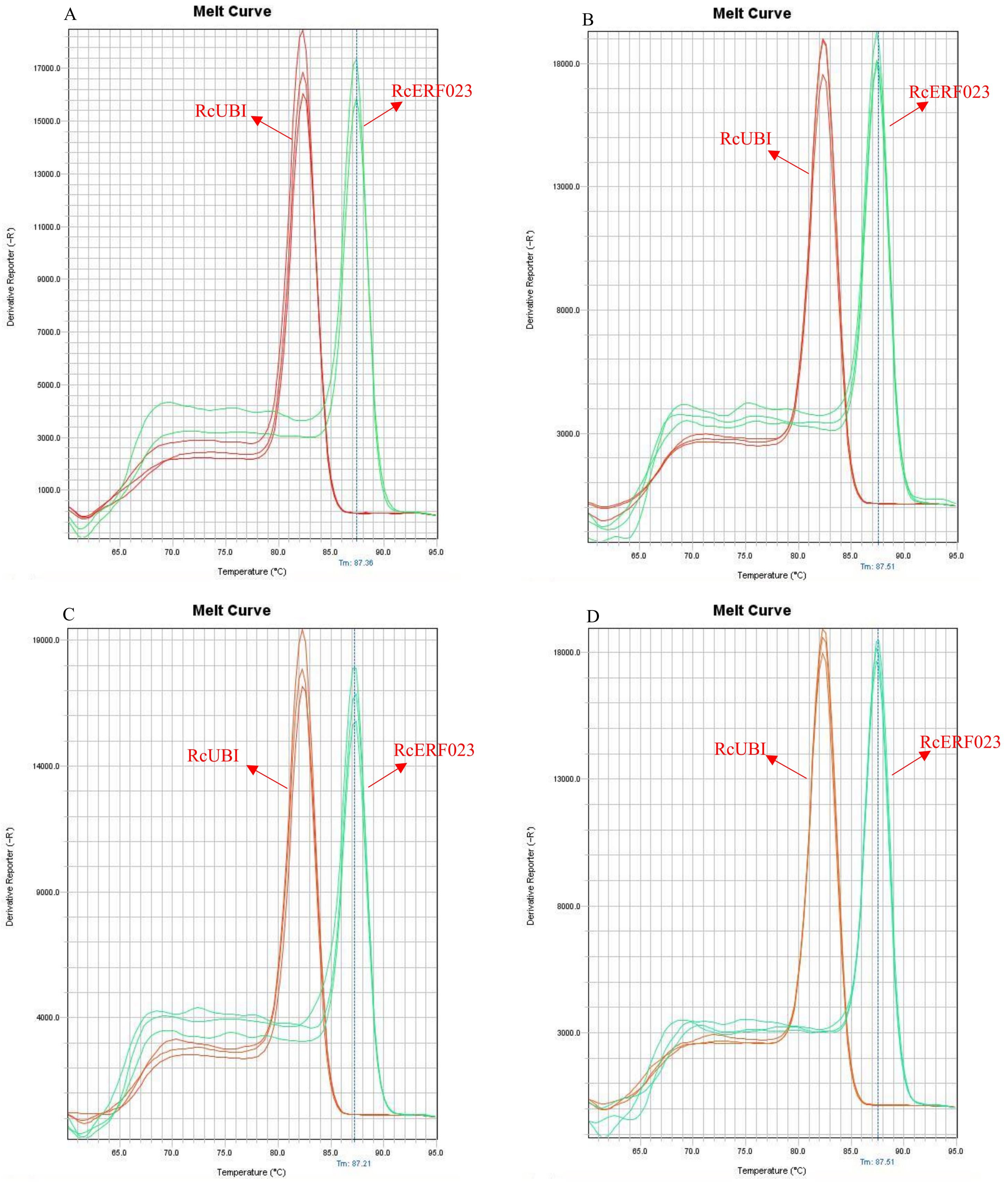

A:30h-PDB B: 30h-BC C: 48h-PDB D:30h-BC

Supplemental Figure S1 Melting curves for each gene and control: RcERF064

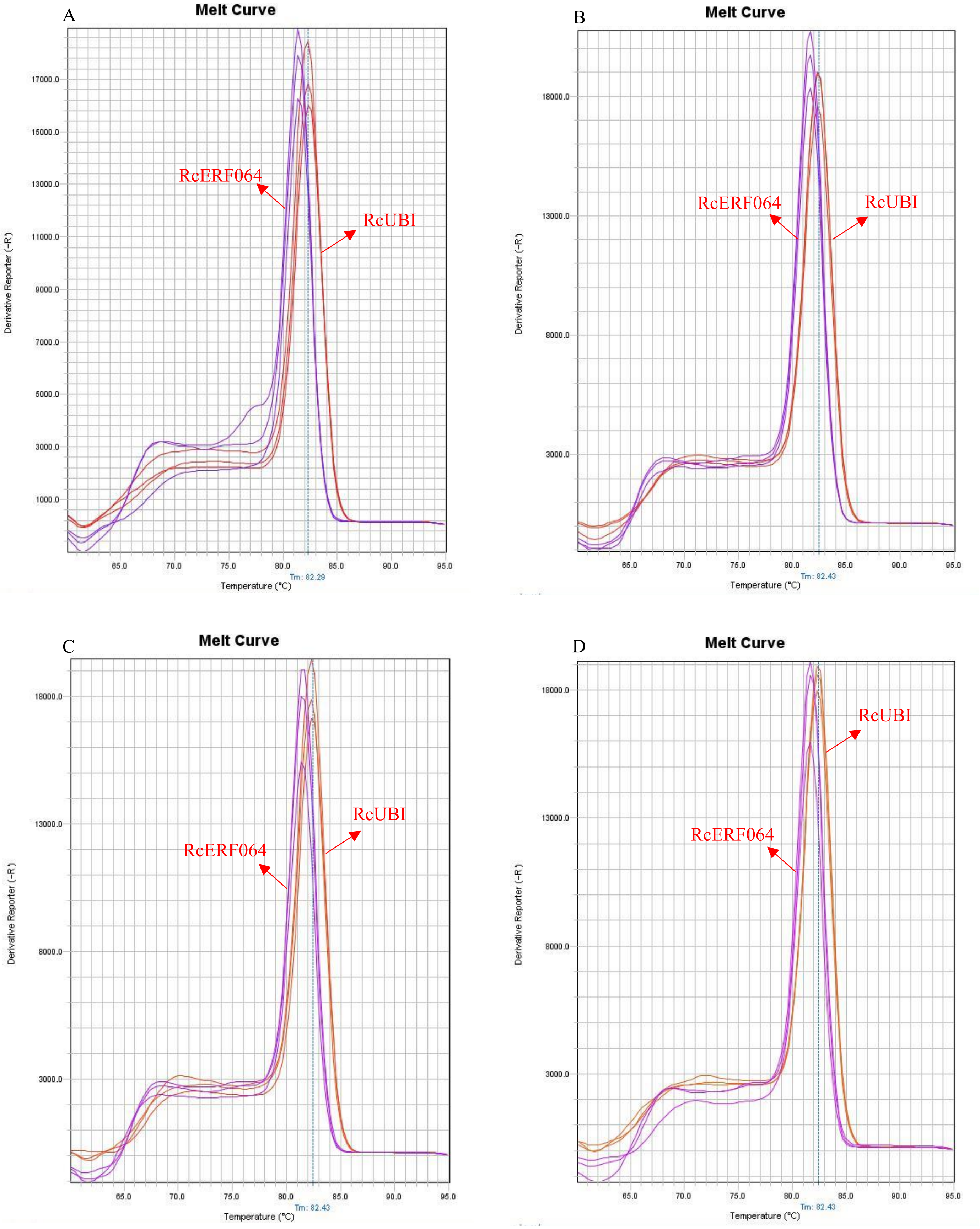

A:30h-PDB B: 30h-BC C: 48h-PDB D:30h-BC

Supplemental Figure S1 Melting curves for each gene and control: RcERF068

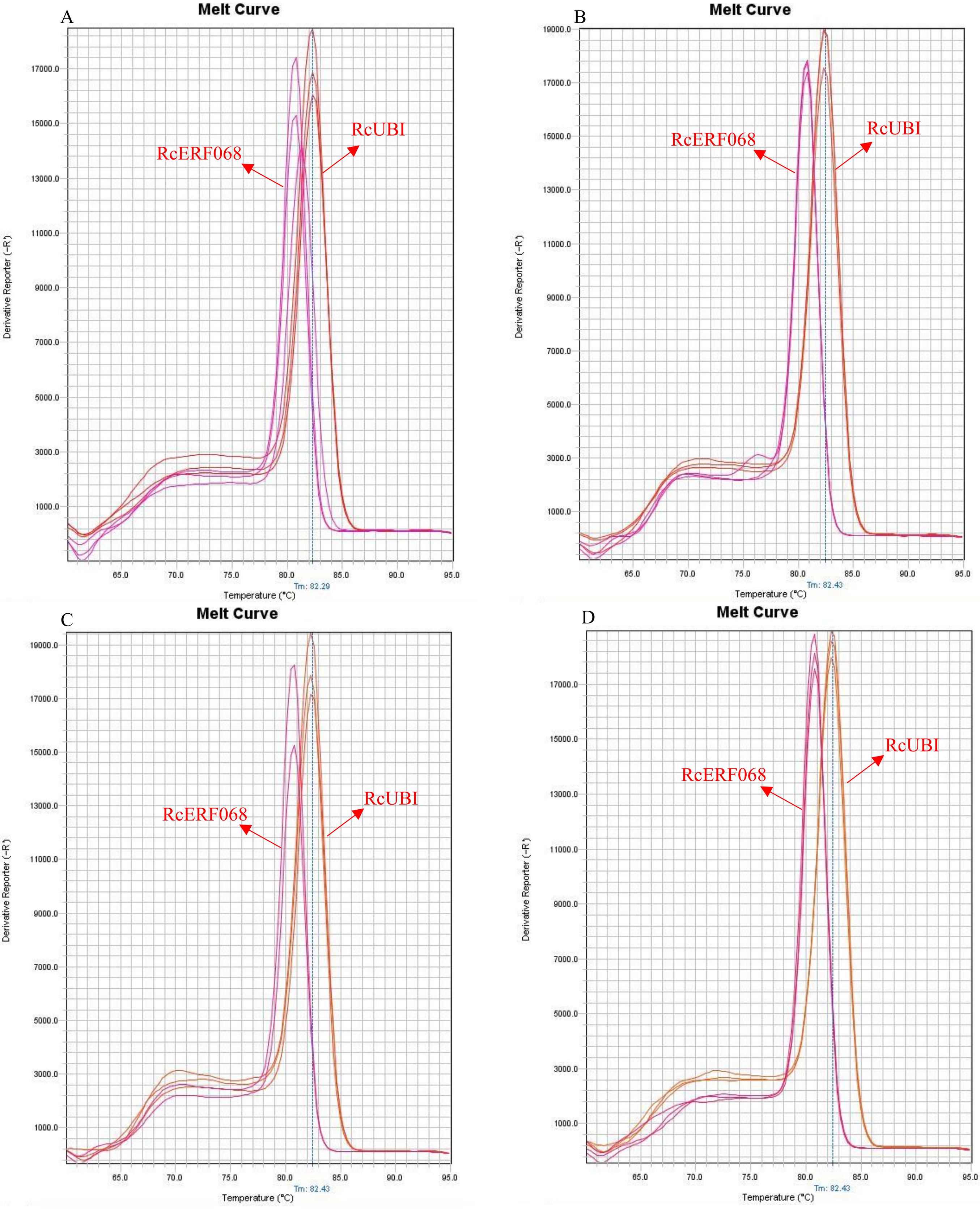

A:30h-PDB B: 30h-BC C: 48h-PDB D:30h-BC

Supplemental Figure S1 Melting curves for each gene and control: RcERF093

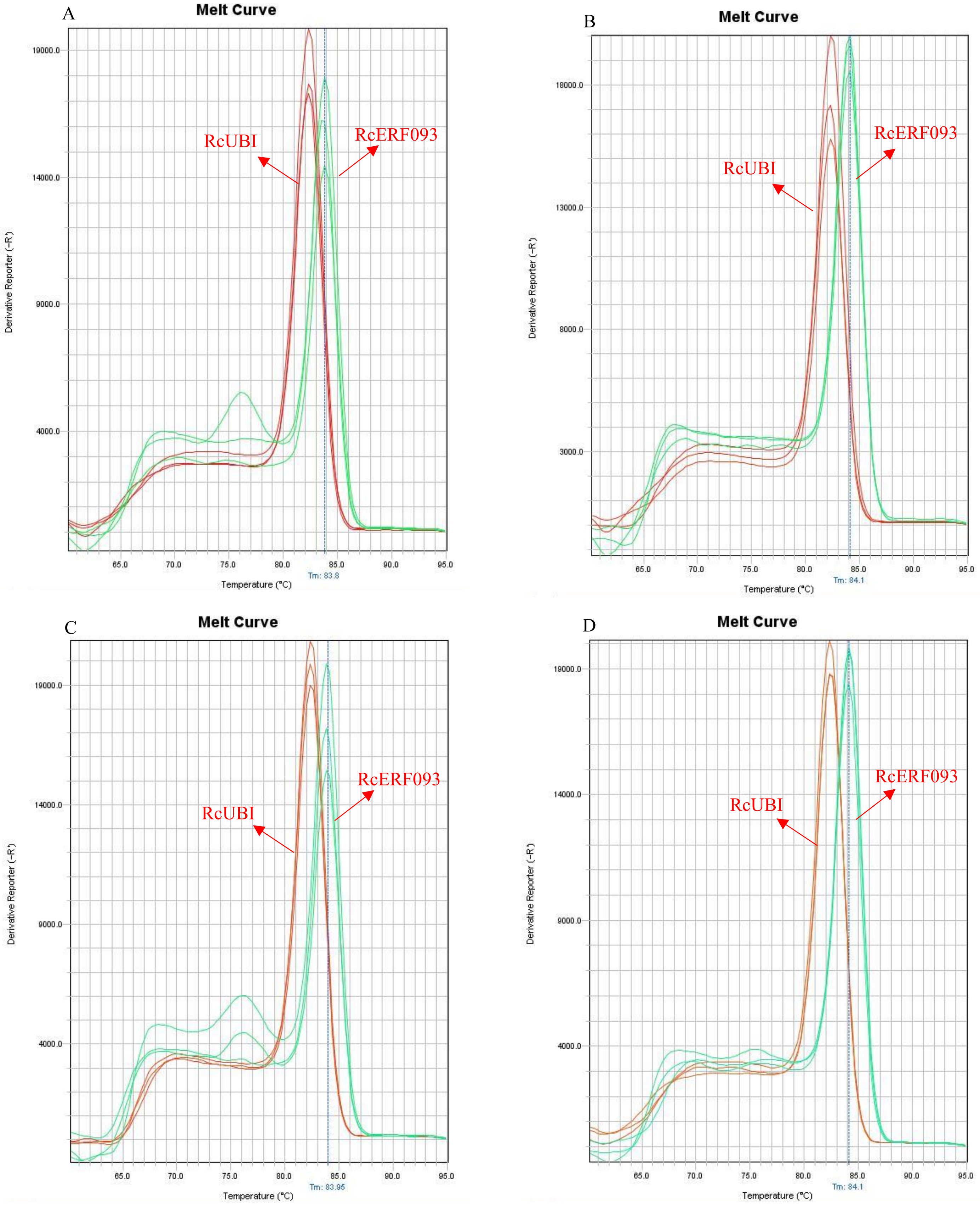

A:30h-PDB B: 30h-BC C: 48h-PDB D:30h-BC

Supplemental Figure S1 Melting curves for each gene and control: RcERF099

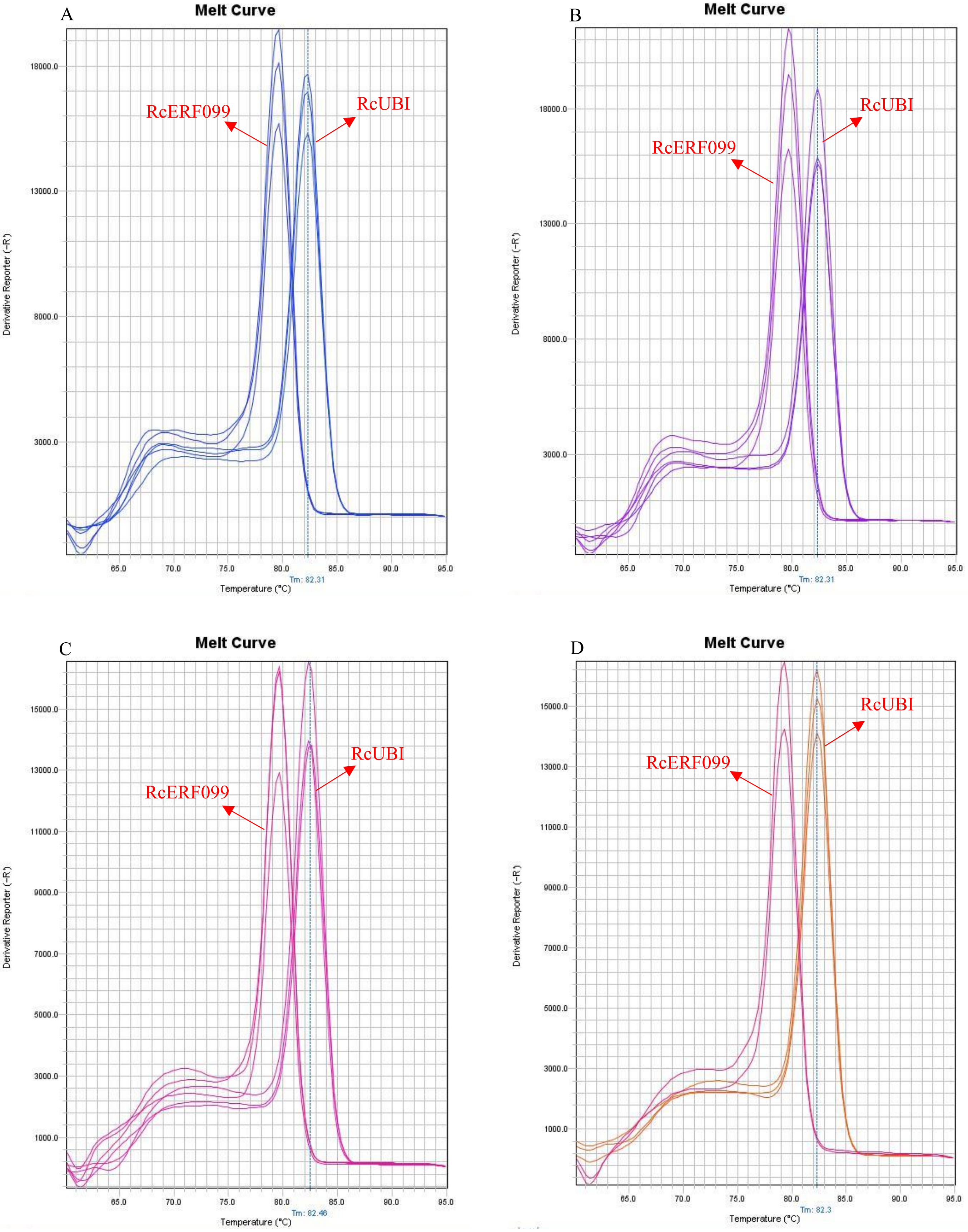

A:30h-PDB B: 30h-BC C: 48h-PDB D:30h-BC

Supplemental Figure S1 Melting curves for each gene and control: RcERF125

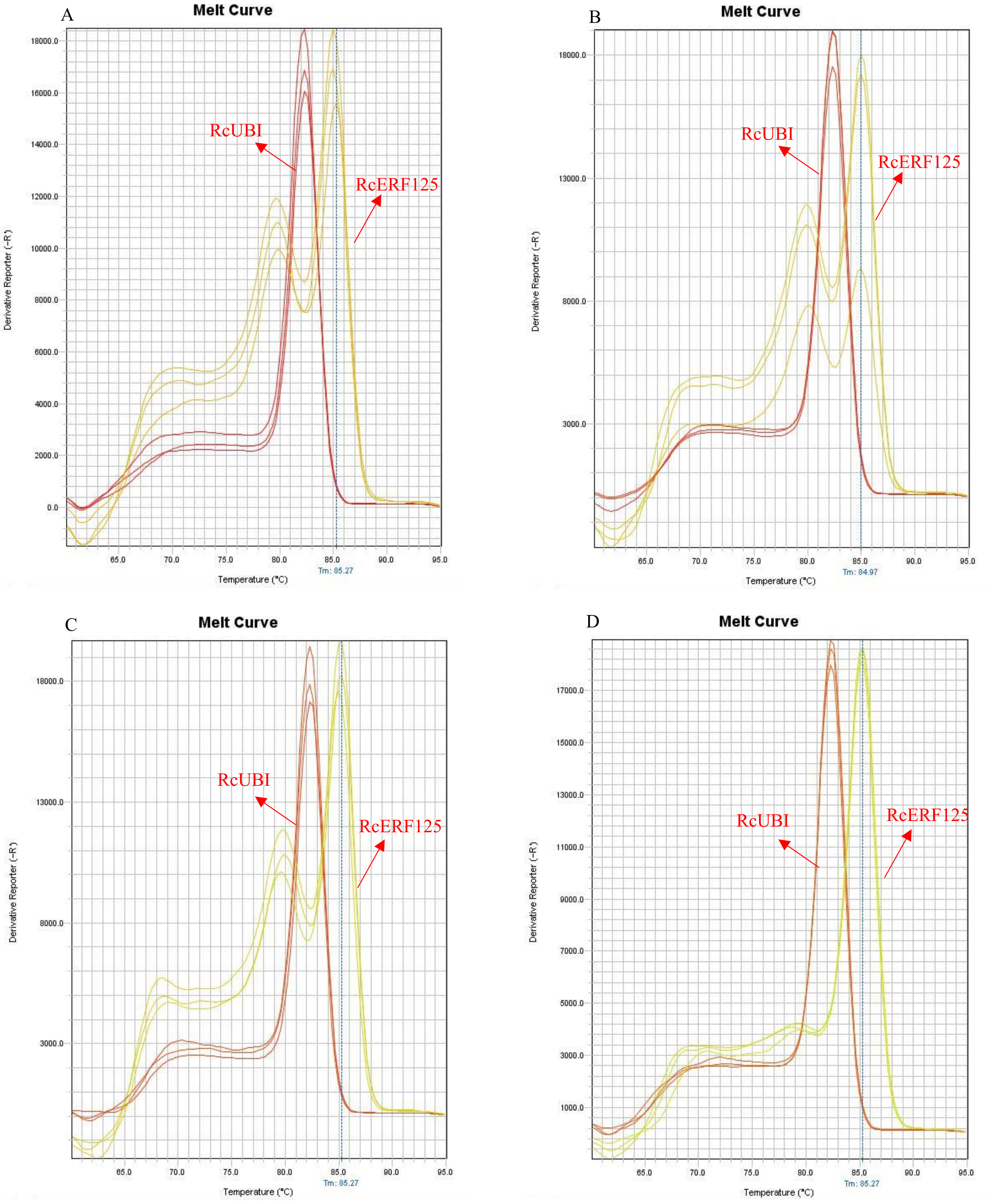

A:30h-PDB B: 30h-BC C: 48h-PDB D:30h-BC
